# Penile length of men attending urology outpatient clinic in Southwest Nigeria

**DOI:** 10.11604/pamj.2021.39.155.21733

**Published:** 2021-06-29

**Authors:** Augustine Oghenewyin Takure

**Affiliations:** 1 Department of Surgery, College of Medicine, University of Ibadan, University College Hospital, Ibadan, Nigeria

**Keywords:** Flaccid, stretched penile length, correlations

## Abstract

Penile length varies worldwide. It is necessary to determine penile length in our region to enable adequate counselling on normal length and reduce avoidable anxiety. Two hundred and seventy-one men were recruited with 97 men in group I and 174 men in group II. Mean ages were 38.4 ± 6.9 (21-50) years and 68 ± 9.1 (51-98) years. In group I, the mean flaccid penile length (FPL)-I and stretched penile length (SPL)-I were 9.8± 2.7 and 10.6± 2.2, mean height 1.7±0.07m and body mass index (BMI) 24.1±3.1 kg/m^2^. While in group II, mean FPL-II and SPL-II were 13.0± 2.9 and 14.1 ± 2.1, mean height was 1.69± 0.07m and BMI 24.3± 3.2 kg/m^2^. There were positive correlations between the SPL-I and heights in group I; (r=0.305, p<0.002); height and FPL-I (r = 0.218, P<0.032). Similarly, in group II positive correlations of FPL-II and SPL-II with height; r = 0.166 P<0.028 and r = 0.183, P<0.015. There was negative correlation SPL-II and BMI-II r-0.224, p<0.003. FLR-II and BMI-II, -0.157, p<0.039. In conclusion, Flaccid and stretched penile length of men < 50 years was smaller than those > 50 years. In both age groups, flaccid and stretched penile lengths correlated with height. In group II, penile lengths correlated negatively with BMI.

## Introduction

Men often complain to their care giver or urologist about their concerns of whether the length of the penis is truly micropenis, that is a stretched penile length less than 7cm. Some are anxious and feel that the penis is small though clinical examination by physicians shows a normal size. It may results in constant obsession to check the penis and may be part of a psychotic disorder. This condition is referred to as the small penis syndrome [1]. On the contrary, women perception of penile length depends on the type of relationship; they prefer width of penis on short term relationship and smaller penis for long term relationship because this reduces the chances of infection and trauma to their vagina. Most women pay less attention to penile length and are more interested in affection [2]. The flaccid and stretch penile length varies from region to region [3-5]. Two different studies in Nigeria reported mean flaccid penile length of 8.6cm and the other stretched penile length of 13.4cm for different age groups [6,7]. The aims of this study were to determine the mean flaccid and stretched penile lengths and to find out if there are correlations between penile length and anthropometry of two age groups of men 20-50 years and 51-99 years with urological problems excluding erectile dysfunction and Peyronie´s disease. It is imperative to establish the true penile length of men in our environment to allay their anxiety.

## Methods

**Study population**: two hundred and seventy-one men with urological disease who presented to a single consultant in a tertiary hospital in southwestern Nigeria over a 3-year period April 2016 to March 2019.

**Study design**: these men were divided into two age groups I; 97 (20-50 years) and group II; 174 (51-98 years). The flaccid penile length (FPL) and stretched penile length (SPL) were prospectively measured in centimeter from the pubic arch to the tip of the glans penis. Their anthropometric parameters were routinely recorded.

**Exclusion criteria**: the men with erectile dysfunction and Peyronie´s disease were excluded.

**Primary objective**: to determine the normal flaccid penile length and stretched penile length in two age groups less than 50 years and greater than 50 years.

**Secondary objectives**: to determine any correlations between the penile length with age, height, weight and body mass index.

**Data analysis**: the data were analyzed using statistical package for social sciences, (version 22.0; Chicago, IL, USA) The penile lengths, age, height and body mass index were analysed using simple statistics of mean and standard deviations of mean. The correlation was determined using Pearson´s coefficient and t-tail significance was place at p< 0.05 at confidence interval of 95%.

**Outcome**: the mean and standard deviations of flaccid penile length and stretched penile length among two age groups in Nigeria. The correlations between penile lengths and anthropometric indices.

**Ethical approval**: this was not obtained because it was an examination that was part of the routine examination of the external genital and does not include use of invasive instruments.

## Results

Two hundred and seventy-one men were recruited with 97 men in group I and 174 men in group II. The mean ages were 38.4 ± 6.9 (21-50) years and 68 ± 9.1 (51-98) years. The t-test between penile length and age groups I and II are statistically significant p< 0.000. Table 1 shows that in group I, the mean flaccid penile length (FPL-I) and stretched penile length (SPL-I) were 9.8 ± 2.7 and 10.6± 2.2, mean height 1.7±0.07m and BMI 24.1 ± 3.1 kg/m^2^. While in group II (51-99years), the mean flaccid penile length (FPL-II) and stretched penile length (SPL-II) were 13.0± 2.9 and 14.1 ± 2.1. The mean height/standard deviation was 1.69± 0.07m whilst the body mass index (BMI) was 24.3± 3.2 kg/m^2^. There were positive correlations between the SPL-I and heights in group I; (r = 0.305, p < 0.002); height and FPL-I (r = 0.218, P < 0.032). Similarly, in group II positive correlations of FPL-II and SPL-II with height; r = 0.166 P < 0.028 and r = 0.183, P < 0.015). Negative correlation SPL-II and BMI-II r-0.224, p < 0.003. FLR-II and BMI-II, -0.157, p<0.039 (Table 2). The mean weights/standard deviation of group I and II are 70.8 ± 10.7 kg and 58.9 ± 11.4 kg respectively.

**Table 1:** paired correlations between group I and group II penile length, height and body mass index

Pairs	Number	Correlation (r)	Significance (p<0.05)
**Penile length & height**			
FPL I & height I	97	0.218	0.032
SPL-I & height I	97	0.305	0.002
FPL-II &height II	174	0.166	0.028
SPL-II & height II	174	0.183	0.015
**Penile length & BMI**			
FPL II & BMI	174	-0.157	0.039
SPL II & BMI	174	-0.224	0.003
**Abbreviations:** FPL, flaccid penile length; SPL, stretched penile length; BMI, body mass index ((kg/m^2^); r, Pearson´s coefficient.			

**Table 2:** comparison of penile length in countries mentioned in the article

Country	References	Sample size	Mean age/SD/range	FPL	SPL	r; p (Ht. &FPL)	r; p (Ht.& SPL)
**Study (Nigeria)**	Group I	97	38.4±6.9(20-50)	9.8±2.7	10.6±2.2	0.218; <0.003	0.305; <0.002
	Group II	174	68±9.1(51-98)	13±2.9	14.1±2.1	0.166 ; <0.003	0.183; <0.015
	Overall	271	57.3±16.4(21-98)	10.3±2.4	13.7±2.5	-	-
**China**	Chen *et al*	5196	-(18-60)	6.5±0.7	12.9±1.2	-	-
**India**	Promodu *et al*	301	31.58(18-60)	8.2±1.4	10.8±1.4	-	-
**Iran**	Mehraban *et al*	1500	29.61(20-40)	-	11.6	-	-
**Italy**	Ponchietti *et al*	3300	-(17-19)	9	12.5	-	-
**Nigeria**	Ajimani *et al*	320	-(17-23)	8.6±0.94	-	-	-
**Nigeria**	Orakwe *et al*	115	42.3(30-60)	-	13.37	-	-
**United Kingdom**	Veale *et al*	15,521	-	9.2±1.6	13.24±1.89	-	-
**Abbreviations:**	SD, standard deviation; F, flaccid; S, stretched, PL, penile length; r, Pearson´s coefficient; p, p value < 0.05						

## Discussion

It is objective to measure the flaccid and stretched penile length of men than the erect penis that is often measured by the men with attendant variations. A systematic review showed that the stretched penile length was identical to the erect penile length [8]. This study divided the men whose penile length were measured into two groups, those less than 50 years and above 50 years, unlike previous study in Nigeria where the age groups were 17 to 23 years and 30 to 65 years [6,7]. Both flaccid penile length and stretched penile length in this study of men less than 50 years was slightly lower than in men above 50 years. The reason for these observed differences in both age groups in this study may be related to the level of testosterone as reported by Boas *et al*. [9]. However, we did not routinely check the serum testosterone of all men who presented with urological problems except those with erectile dysfunction who were excluded. Future study would address any correlation between penile length and the serum testosterone of normal subjects without urological symptoms. This study showed a longer flaccid and stretched penile length of the two groups compared to the previous studies in Nigeria that studied these lengths separately [6,7].

The overall mean flaccid penile length (10.3cm) in this study was slightly more than that reported by Veale *et al*. in a systematic review in the United Kingdom [8]. However, both the mean flaccid penile length (6.5cm) and stretched penile length (12.9cm) of Chinese attending urology clinic were smaller compared to overall mean flaccid and stretched penile lengths in this study [10]. Similarly, the overall stretched penile length in this study was comparable to 13.2 cm in the United Kingdom study [8]. Among Indians with similar age range to group I men in our study, their mean flaccid and stretched penile length were similar [4]. The reason for this similarity is not clear particularly when the average Indians are vegetarians whilst their Nigerian counterpart are non-vegetarians. We may need to explore assessing their testicular volumes and serum testosterone for any similarity. The stretched penile length (10.6cm) of Nigerian men less than 50 years was lower when compared to studies with age less than 40 years; Iranian men (11.59cm) and Italian men (12.5cm) [3,5]. In this study, there was positive correlations between flaccid and stretched penile lengths of both groups with heights. These findings are comparable to studies among Iranians, Italians, and British men in the United Kingdom [3,5,8]. There was a negative correlation between both flaccid and stretched penile lengths with body mass index as among Indians [7].

## Conclusion

The flaccid and stretched penile length of men < 50 years was smaller than those > 50 years. In both age groups, flaccid and stretched penile lengths correlated with height, however, in group II, penile lengths correlated negatively to BMI.

### What is known about this topic

- The penile length of various race, and countries;
- The penile length of two studies from Nigeria at different unrepresentative age groups.

### What this study adds

- The mean adult penile length of two age groups I (20-50 years) and group II (51-98);
- The correlation of penile length with height and body mass index in our environment not documented in previous studies.
